# Regulation of Ferroptosis Pathway by Ubiquitination

**DOI:** 10.3389/fcell.2021.699304

**Published:** 2021-08-13

**Authors:** Xinbo Wang, Yanjin Wang, Zan Li, Jieling Qin, Ping Wang

**Affiliations:** Tongji University Cancer Center, Shanghai Tenth People’s Hospital, School of Medicine, Tongji University, Shanghai, China

**Keywords:** ferroptosis, ubiquitination, lipid peroxidation, cell metabolism, cancer therapy

## Abstract

Ferroptosis is an iron-dependent form of programmed cell death, which plays crucial roles in tumorigenesis, ischemia–reperfusion injury and various human degenerative diseases. Ferroptosis is characterized by aberrant iron and lipid metabolisms. Mechanistically, excess of catalytic iron is capable of triggering lipid peroxidation followed by Fenton reaction to induce ferroptosis. The induction of ferroptosis can be inhibited by sufficient glutathione (GSH) synthesis via system Xc^–^ transporter-mediated cystine uptake. Therefore, induction of ferroptosis by inhibition of cystine uptake or dampening of GSH synthesis has been considered as a novel strategy for cancer therapy, while reversal of ferroptotic effect is able to delay progression of diverse disorders, such as cardiopathy, steatohepatitis, and acute kidney injury. The ubiquitin (Ub)–proteasome pathway (UPP) dominates the majority of intracellular protein degradation by coupling Ub molecules to the lysine residues of protein substrate, which is subsequently recognized by the 26S proteasome for degradation. Ubiquitination is crucially involved in a variety of physiological and pathological processes. Modulation of ubiquitination system has been exhibited to be a potential strategy for cancer treatment. Currently, more and more emerged evidence has demonstrated that ubiquitous modification is involved in ferroptosis and dominates the vulnerability to ferroptosis in multiple types of cancer. In this review, we will summarize the current findings of ferroptosis surrounding the viewpoint of ubiquitination regulation. Furthermore, we also highlight the potential effect of ubiquitination modulation on the perspective of ferroptosis-targeted cancer therapy.

## Introduction

All living organisms have been refined by the natural selection during the evolution. A sophisticated and unique reproduction system has been evolved in various species to ensure a sustained anagenesis (Bedoui et al., 2020; Rothlin et al., 2020; Koren and Fuchs, 2021). Cell suicide, namely, programmed cell death, includes apoptosis (Taylor et al., 2008), necroptosis (Zong and Thompson, 2006), ferroptosis (Green, 2019), and pyroptosis (Nagata, 2018). Ferroptosis, which is a novel type of programmed cell death, is characterized by a dysregulated iron metabolism and accumulation of lipid peroxides (Stockwell et al., 2017). Ferroptosis differs from other types of cell death such as apoptosis and necrosis. It features the alteration of mitochondria and aberrant accumulation of excessive iron as well as loss of cysteine–glutathione–GPX4 axis, a major cellular antioxidant system (Tang D. et al., 2021). While catalytic iron is indispensably involved in cell growth of all organisms, it ensures the essential function of vital enzymes encompassing oxygen transport, ATP generation, and DNA synthesis (Silva and Faustino, 2015). However, excessive iron can also impede cells by induction of Fenton reaction, leading to various DNA damages and even cell death (Eid et al., 2017). Therefore, maintaining an appropriate labile iron is critical for cell viability. Cells ongoing ferroptosis, however, show a dysregulated iron metabolism displaying ceaseless iron intake and retention. Eventually, a mass of catalytic iron assembled in the cytosol and other organelles contributes to lipid peroxidation, which will lead to ferroptosis (Conrad and Proneth, 2020). Although the research contribution of ferroptosis has been more and more fruitful, the involvement of post-translational regulation in ferroptosis has been largely unknown yet.

**FIGURE 1:**
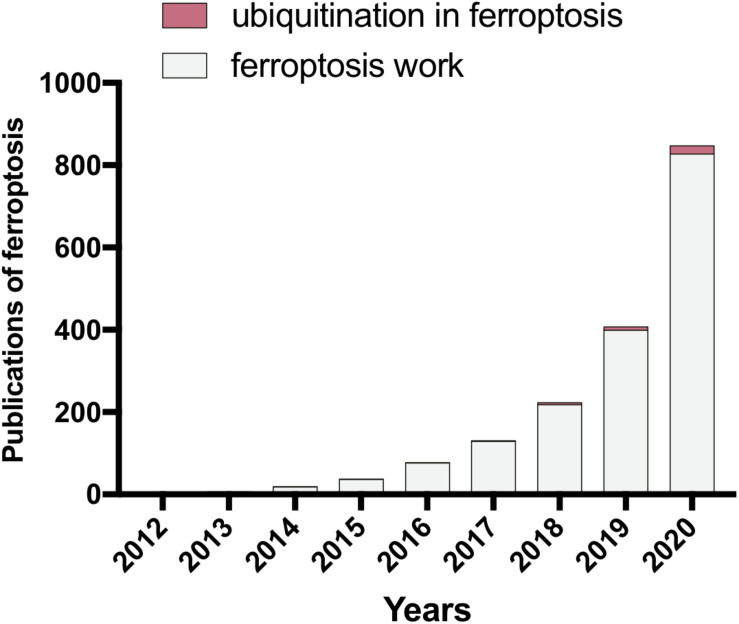
The publications of ferroptosis study in the last decade (PubMed). Since 2012, the contributions of ferroptosis research have been dramatically increased throughout the world. The importance of ubiquitination has been gradually uncovered in the area of ferroptosis research. However, further studies are still required to elucidate more details of the connection between ubiquitination and ferroptosis.

In living cells, the ubiquitin (Ub)–proteasome pathway (UPP) dominates the majority of intracellular protein degradation by coupling Ub molecules to the lysine residues of protein substrate, which is subsequently recognized by the 26S proteasome for degradation (Lu et al., 2021). Dysregulated ubiquitination has been implicated in neurological diseases and tumorigenesis (Bard et al., 2018; Lu et al., 2021). Recently, emerged evidence has emphasized the crucial roles of ubiquitination in ferroptosis regulation. Although the regulation of ubiquitous pathway in cells ongoing ferroptosis remains elusive, the crosstalk between ubiquitous modulation and ferroptosis has captured the more and more imagination of researchers (Figure 1). Herein, we summarize the progression of ubiquitination regulation in ferroptosis in recent years. Furthermore, we look into the distance to the development trend of ferroptosis in the clinical application of cancer therapy by targeting ubiquitous regulation.

## The Hallmarks of Ferroptosis

Although the increased iron supply and accelerated lipid production satisfy the demand of cancer cells to boost cancer cell division and spreading, excessive iron will greatly accelerate lipid peroxidation, which consequently gives rise to higher vulnerability to ferroptosis. Apart from other types of cell death, ferroptosis appears to show an iron-addiction phenotype accompanied by a lipid peroxidation phenomenon. Herein, we will discuss the recent findings related to ferroptosis surrounding these points.

### Alteration of Iron Metabolism

**FIGURE 2:**
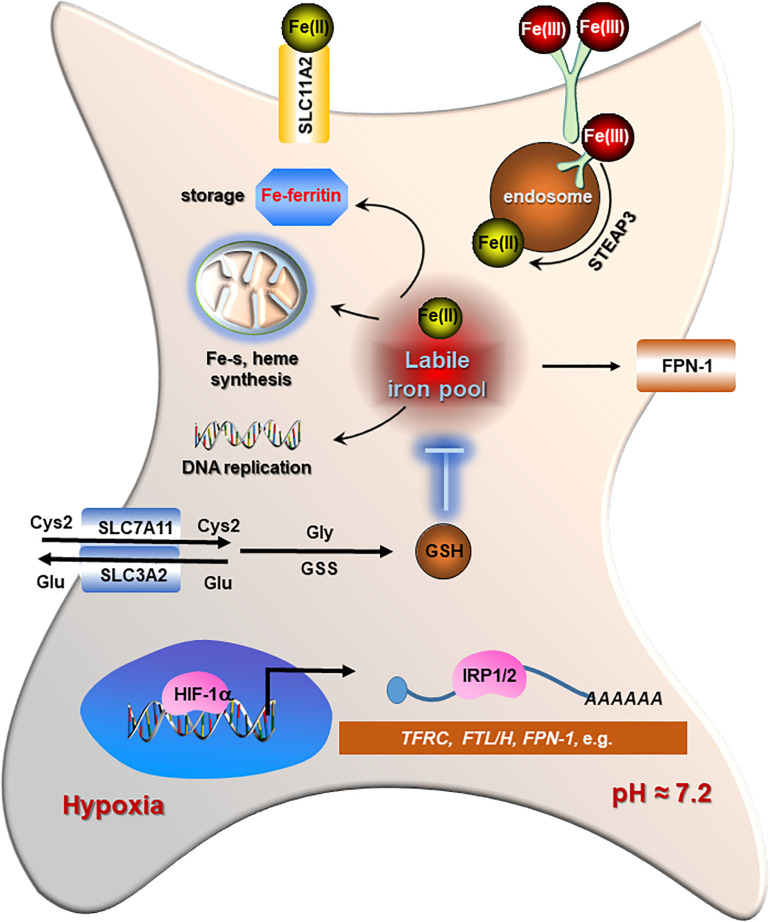
The regulations of iron metabolism and redox homeostasis in cancer cells. Cancer cells display higher iron transporting, storage, and bioavailability as well as increased levels of glutathione (GSH) and GPX4 for detoxification in contrast to normal cells. Iron regulatory proteins (IRP1/2) play a central role in maintaining an adequate iron homeostasis in cancer cells by regulating the stability of each mRNA differentially [increasing for transferrin receptor 1 (TFRC) and SLC11A2, while decreasing for light chain (FTL)/H protecting cells]. The classical marker of hypoxia, HIF-1α, also supports the stabilization of TFRC, IRP1/2, as well as FTL/H to promote both iron absorption and availability in cancer cells.

Iron is one of the most abundant elements on Earth and indispensably involved in cell growth of all organisms. It ensures the essential function of vital enzymes encompassing oxygen transport, ATP generation, and DNA synthesis (Sheftel et al., 2012). Iron possesses unpaired electrons, exhibiting a wide range of oxidation states that contribute to its versatile participant in redox reactions (Barton et al., 2019), which endow iron with crucial roles in maintaining biological activities, such as cell division, metabolism, and growth (Torti et al., 2018). Therefore, maintaining an appropriate labile iron is critical for cell viability. Cells ongoing ferroptosis, however, show a dysregulated iron metabolism displaying ceaseless iron intake and retention. Briefly, intracellular iron acquisition is predominantly mediated by transferrin receptor 1 (TFRC) that engages in uptake of transferrin-bound Fe (III) and cooperates with clathrin-mediated endocytosis (Kawabata, 2019). Then the absorbed iron is released into acidic endosomes where the Fe (III) is reduced to Fe (II) status by the ferriductase enzyme STEAP3 (Ohgami et al., 2005). Then, Fe (II) is released from endosome to cytosol by divalent metal transporter 1 (DMT1), which is also involved in Fe (II) and other ions such as cadmium (Ca^2+^), copper (Cu^2+^), and zinc (Zn^2+^) uptake across the plasma membrane (Illing et al., 2012). Iron storage is primarily conducted by ferritin protein complex, which comprises heavy chain (FTH) and light chain (FTL) protecting cells against reactive oxygen species (ROS) (Vidal et al., 2008; Zhang et al., 2009). Ferroportin (FPN1), the only known iron exporter, enables iron exporting across the plasma membrane (Ward and Kaplan, 2012). The catalytic iron transiently assembled in the cytosol constitutes a labile-iron pool (LIP) serving as a crossroad of intracellular iron trafficking (Kakhlon and Cabantchik, 2002; Figure 2).

Iron addiction, which is commonly existing in most malignancies, has been revealed as a potential risk of ferroptosis (Basuli et al., 2017; Li et al., 2019). The rapid consumption of iron fulfills the needs of aggressive behaviors including higher proliferation, metastasis, and invasion in tumors (Hann et al., 1988). Furthermore, the enrichment of catalytic iron in tumor cells can be further enhanced by hypoxia. The increased levels of iron transporters (TFRC and DMT1) and iron regulatory protein 2 (IRP2) have been uncovered in response to activation of HIF-1 accompanied by stabilization of iron-storage proteins (FTL/H) (Hanson et al., 1999; Tacchini et al., 1999; Qian et al., 2011; Huang et al., 2014; Li et al., 2019). These evidence suggests that iron addiction is favored by cancer cells. However, it may also potentially render malignancies to be highly vulnerable to iron-induced cytotoxicity contributing to ferroptosis.

### Lipid Peroxidation in Ferroptosis

**FIGURE 3:**
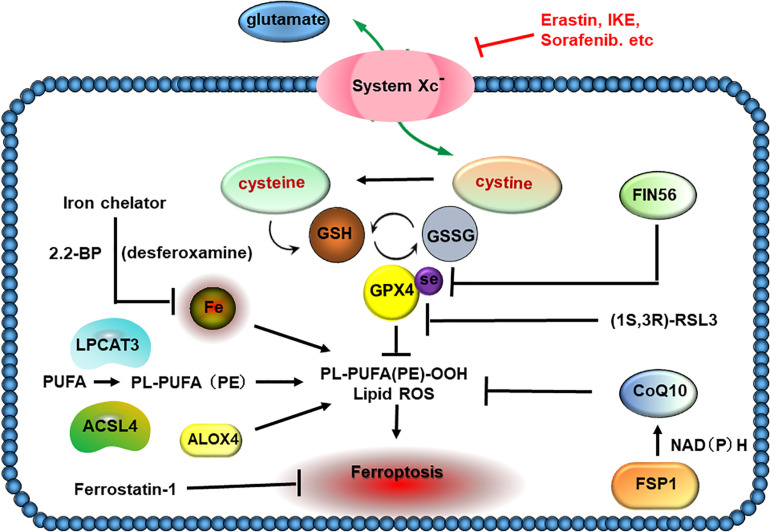
Lipid peroxidation in ferroptosis. The increased absorption of cysteine in tumor cells is utilized to build up the cysteine–glutathione (GSH)–GPX4 axis, which plays a crucial role in detoxifying cellular oxidants and evading ferroptosis. Acyl-CoA synthetase long-chain family member 4 (ACSL4) associates with lysophosphatidylcholine acyltransferase 3 (LPCTA3) to incorporate polyunsaturated fatty acids (PUFAs) into PL-PUFA (PE), which shows higher susceptibility to peroxidation and ferroptosis. The catalytic iron inside cells appears to be the source of Fenton chemistry, which creates hydroxyl and peroxyl radicals capturing hydrogen atoms from PUFAs and triggering peroxidation of PL-PUFA. FSP1, a novel finding of ferroptosis suppressor, protects cells against ferroptosis by catalyzing the regeneration of CoQ10 using NAD(P)H. FIN56 induces ferroptosis by promoting the GPX4 degradation and lowering the CoQ10 amount.

The catalytic radicals induced by excessive iron will attack electrons from the lipids localized in the plasma and organelle membranes (Chen et al., 2021a; Yan et al., 2021). Lipid peroxidation can be caused by either non-enzymatic iron-catalyzed form or enzymatic generation of signals (Conrad and Pratt, 2019). Acyl-CoA synthetase long-chain family member 4 (ACSL4) dominates the catalyzing reaction, which converts arachidonoyl (AA) or adrenoyl (AdA) into AA or AdA acyl-CoA derivatives (AA-CoA or AdA-CoA). Both AA-CoA and AdA-CoA will be esterified by lysophosphatidylcholine acyltransferase 3 (LPCTA3) to produce phosphati-dylethanolamines (AA-PE and AdA-PE). Subsequently, AA-PE and AdA-PE will be oxidized by 15-lipoxygenase (ALOX15), which is iron-containing dioxygenase that catalyzes the hydrogen abstraction of polyunsaturated fatty acid (PUFA) to generate lipid hydroperoxides and induce ferroptosis (Mashima and Okuyama, 2015). Importantly, iron also exerts roles in oxidative cleaving of 15-hydroperoxy-AA-PE (HOO-AA-PE), which is able to react with protein targets to induce plasma membrane disruption (Mashima and Okuyama, 2015; Figure 3).

### Main Regulators of Ferroptosis

**FIGURE 4:**
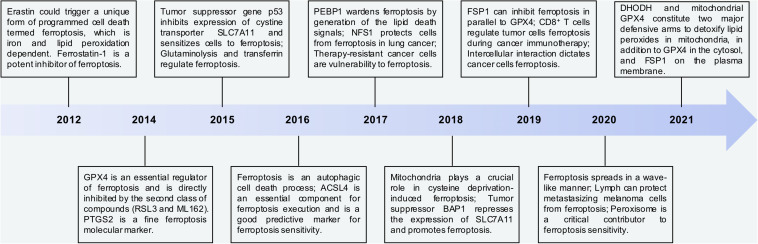
The key points in discovery and research history of ferroptosis. Time line of key findings in ferroptosis research. Ferroptosis was firstly defined in 2012. In this decade, a great number of efforts have contributed to the progression of ferroptosis work. Overall, DHODH and mitochondrial GPX4 are two major defensive arms to detoxify lipid peroxides in the mitochondria, in addition to FSP1 on the plasma membrane and GPX4 in the cytosol.

In the recent decade, a great number of efforts have been contributed to the progression of ferroptosis work. Thereby, we summarize the key findings related to ferroptosis since 2012 (Figure 4). Moreover, we emphasize the studies about ubiquitination modification in ferroptosis according to the recent findings.

Dixon et al. (2012) (Brent R. Stockwell Lab) have proposed that erastin, a small-molecule compound, can efficiently kill cancer cells by induction of iron-dependent cell death, named ferroptosis. Apparently, this type of cell death is morphologically and biochemically distinct from other types of cell death (Stockwell et al., 2017). Mechanistically, erastin inhibits cystine uptake via the cystine/glutamate Xc^–^ antiporter, which has been found overexpressed in many types of cancer. Disruption of cysteine–GSH–GPX4 remarkably triggers ferroptosis in cancer cells. Solute carrier family 7 member 11 (SLC7A11), one of the components constituting Xc^–^ antiporter, is transcriptionally regulated by nuclear factor, erythroid 2-like 2 (NRF2) and ATF3/4 (Ye et al., 2014; Ogiwara et al., 2019), indicating the key role of cystine metabolism and redox balance in ferroptosis (Chu et al., 2019; Zhang Y. et al., 2021).

Additionally, further investigation has indicated that this type of cell death is accompanied by induction of lipid peroxidation and aberrant morphology of the mitochondria. Moreover, iron chelator also shows effective inhibitory effect on erastin-induced cell lethality (Chen P.H. et al., 2021; Zeng et al., 2021). Besides, ferrostatin-1, a specific lipid ROS scavenger, has been found to be a potent inhibitor of ferroptosis in cancer cells, which is hereafter widely used in ferroptosis research (Lee et al., 2020; Hong et al., 2021). As Xc^–^ antiporter is an essential factor dictating ferroptosis susceptibility (Lang et al., 2019), its corresponding ubiquitination regulators [E3 ubiquitin ligases and deubiquitylating enzymes (DUBs)] are supposed to be theoretically important for ferroptosis regulation.

In parallel with SLC7A11, selenium-containing GPX4 is another key protein with a potent role in blocking ferroptosis. Yang et al. (2014) have discovered that GPX4 is the target of ferroptosis inducing compounds RSL3 and ML162, thus, revealing GPX4 as an essential protector against ferroptosis. In this study, the authors have also identified that *PTGS2*, a gene encoding cyclooxygenase-2 (COX-2), was the utmost expression gene in response to RSL3 treatment (Wu et al., 2019; Li et al., 2020; Yi et al., 2020). It should be noted that degradation of GPX4 has been revealed in cells ongoing ferroptosis, which is irrespective of GPX4 activity inhibition (Chen et al., 2021b), suggesting that ubiquitination modification of GPX4 degradation is supposed to be existing.

Inactivation of the p53 has been found in many types of cancer (Hafner et al., 2019; Levine, 2020). p53 has been considered as a potent tumor suppressor due to multiple roles in cell cycle arrest, DNA damage, apoptosis, and senescence (Kakhlon and Cabantchik, 2002; Jiang et al., 2015; Boutelle and Attardi, 2021). Unexpectedly, it has been reported that p53 inhibits cystine uptake and sensitizes cells to ferroptosis by repressing the expression of SLC7A11 (Jiang et al., 2015), thus, uncovering a novel role of p53 in ferroptosis. Notably, p53 is tightly regulated by both E3 ubiquitin ligases (MDM2, TRIM69, UBE2T, RBCK1, COP1, and CHIP) and DUBs (USP7, USP3, USP11, USP15, USP49, OTUD1, and OTUD5), suggesting a crosslinking between ubiquitination and p53-mediated ferroptosis (Liu Y. et al., 2019).

Nutrient availability dictates the cell survival and proliferation rate, especially in tumor cells (Hoxhaj and Manning, 2020; Sukjoi et al., 2021). Long-time deprivation of amino acids, glucose, or growth factors are able to result in cell death, which is considered as a passive death process (Tummers and Green, 2017; Hayes et al., 2020). It has been indicated that ferroptosis is associated with serum supplement upon amino acid starvation. Both iron carrier protein transferrin and amino acid glutamine have been demonstrated as ferroptosis inducers (Gao et al., 2015). Moreover, a crosstalk among different types of cell death has been well demonstrated (Nikoletopoulou et al., 2013; Kasprowska-Liśkiewicz, 2017; Frank and Vince, 2019; Snyder and Oberst, 2021). In addition, Gao et al. (2016) have found that ferroptosis is an autophagic cell death process due to the degradation of iron storage protein ferritin (FTH1) mediated by nuclear receptor coactivator 4 (NCOA4), referred to as ferritinophagy. Consequently, the resultant ferrous iron liberated from the breakdown of ferritin amplifies the labile iron pool in cytosol and results in a large accumulation of ROS, eventually triggering lipid peroxidation and ferroptosis (Gao et al., 2016; Hou et al., 2016). Since ubiquitination has been shown to largely participate in amino acid metabolism and autophagy regulation (Kwon and Ciechanover, 2017; Harper et al., 2018; Senft et al., 2018), thus, we propose that ubiquitination is potentially closely related to the ferroptosis process. Additionally, whether ubiquitination occurs in iron-related protein, such as TFRC and FTH1, needs further investigation.

Lipid peroxidation is a hallmark of ferroptosis (Zou et al., 2020a), and PUFA biosynthesis dictates ferroptosis sensitivity (Yang and Stockwell, 2016; Wu et al., 2020). Doll et al. (2017) have uncovered acyl-CoA synthetase long-chain family member 4 (ACSL4) as a crucial player in ferroptosis execution. Moreover, the ACSL4 expression is indicative of ferroptosis confirmed by several studies (Doll et al., 2017; Bersuker et al., 2019; Zou et al.,2020a,b). ACSL4 is responsible for the esterification of CoA to long-chain PUFAs, a key step involved in ferroptosis (Doll et al., 2017). Arachidonic acid has been indicated to promote ACSL4 ubiquitination and proteasomal degradation (Kan et al., 2014). Further investigation found that p115, the vesicular trafficking protein, may be involved in regulation of ACSL4 degradation. However, the specific E3 ubiquitin ligases and DUBs for ACSL4 remained to be elucidated (Sen et al., 2020). The oxygenation of PUFAs by ALOX15 has been found involved in ferroptosis execution (Li et al., 2018). Wenzel et al. have discovered that phosphatidylethanolamine-binding protein 1 (PEBP1), a scaffold protein inhibitor of protein kinase cascades, complexes with ALOX15 and changes its substrate competence to generate hydroperoxy-PE to promote ferroptosis (Wenzel et al., 2017). Further studies are needed to elucidate whether there are some post-translational modifications on ALOX15 and PEBP1 to affect ferroptosis sensitivity.

As mentioned above, ferroptosis is featured by dramatic morphological changes of mitochondria, including mitochondrial fragmentation and cristae enlargement (Del Re et al., 2019; Bock and Tait, 2020), whereas the underlying mechanism is largely unknown for a long time. Some studies have shown that the mitochondria play a crucial role in cysteine deprivation-induced ferroptosis. Mechanistically, the mitochondrial tricarboxylic acid (TCA) cycle and electron transport chain can promote cysteine deprivation-induced ferroptosis by serving as the major source for cellular lipid peroxide production (Gao et al., 2019). It will be interesting to demonstrate whether ubiquitination is involved in mitochondrial alteration in cells ongoing ferroptosis, as the clearance of dysfunctional mitochondria (known as mitophagy) requires Parkin, the E3 ubiquitin ligase that promotes ubiquitination of mitochondrial proteins (Ashrafi and Schwarz, 2013; Pickrell and Youle, 2015; Ravanelli et al., 2020; Song et al., 2021a).

GPX4 is regarded as an effective suppressor of ferroptosis. However, some cancer cells that expressed a low level of GPX4 strongly confers resistance to ferroptosis, suggesting that additional ferroptosis suppressors are supposed to be existing. By using synthetic lethal CRISPR–Cas9 (clustered regularly interspaced short palindromic repeats–Cas9) screening and an overexpression cloning approach, Bersuker et al. (2019) and Doll et al. (2019) have identified apoptosis-inducing factor mitochondria-associated 2 (AIFM2, also known as FSP1) as a key component of CoQ antioxidant system that acts in parallel with the canonical GPX4 pathway. The FSP1–CoQ10–NADPH pathway exists as a stand-alone parallel system, which coordinates GPX4 and GSH to suppress phospholipid peroxidation and ferroptosis. Notably, FSP1 has been shown to be highly ubiquitinated (Hornbeck et al., 2015), suggesting ubiquitination modification and corresponding E3 ligases or DUBs regulating FSP1 may play vital roles in dictating ferroptosis sensitivity.

Cancer immunotherapy can enhance the effector function of CD8^+^ T cells in the tumor microenvironment. It is traditionally considered that CD8^+^ T cells enable tumor cell apoptosis. Unexpectedly, Wang W. et al. (2019) have found that immunotherapy-activated CD8^+^ T cells can enhance lipid peroxidation in tumor cells, which contributes to the antitumor efficacy of immunotherapy. Mechanistically, interferon gamma (IFNγ) released from CD8^+^ T cells enables the reduced expression of SLC7A11. Ionizing radiation (IR) induces substantial tumor cell death and is, thus, widely used in cancer treatment. Similarly, it has been showed that IR promotes ferroptosis in cancer cells (Lang et al., 2019), which is associated with elevation of ACSL4 expression, resulting in amplified lipid peroxidation (Lei et al., 2020). Therefore, ubiquitination of both antiporter system Xc^–^ and ACSL4 may influence ferroptosis and may be an effective strategy for immune- and radiotherapies.

Ferroptosis occurs not only in cell-autonomous mechanism; cell density has been revealed to impact on ferroptosis susceptibility via the Hippo signaling pathway signaling axis. In epithelial cells, E-cadherin enables the inhibitory effect on ferroptosis by activating the intracellular Merlin (NF2) and Hippo signaling pathway. Antagonizing this signaling axis allows the transcriptional coactivator yes-associated protein (YAP) to promote ferroptosis by upregulating expression levels of both ACSL4 and TFRC (Wu et al., 2019). Consistently, another study has found that s-phase kinase-associated protein 2 (SKP2), an E3 ubiquitin ligase, is a direct target of YAP-regulating ferroptosis (Yang et al., 2021). PDZ-binding motif (TAZ) is also considered as a regulator of ferroptosis in renal and ovarian cancer cells (Yang et al., 2019c). More recently, it has been suggested that ferroptosis signal appears to be spread through cell populations in a wave-like manner, resulting in a distinct spatiotemporal pattern of cell death (Riegman et al., 2020). Ubiquitination is potentially essential for cell interaction-mediated ferroptosis since it plays an important role in the Hippo pathway. For example, β-TrCP is the well-known E3 ligase of YAP/TAZ, which promotes the reduction of YAP/TAZ, while E3 ligase ITCH targets LATS1/2 for degradation. It will be interesting to explore the potential roles of these E3 ubiquitin ligases in regulating ferroptosis (Kim and Jho, 2018; Deng et al., 2020).

The reason why cancer cells are always carried in the lymphatic system prior to circulation in the blood is largely unknown. A recent study has found that melanoma cells in lymph can experience less oxidative stress and induce more metastasis foci than those in blood due to higher levels of GSH and oleic acid, which attenuate oxidative stress and ferroptosis. Moreover, oleic acid protects melanoma cells against ferroptosis in an ACSL3-dependent manner and increases the capacity to form metastatic tumors (Ubellacker et al., 2020). Thus, ubiquitination may also regulate ferroptosis during metastasis of cancer cells by regulating ACSL3 stability and oleic acid metabolism.

## Ubiquitination Regulation in Ferroptosis

**FIGURE 5:**
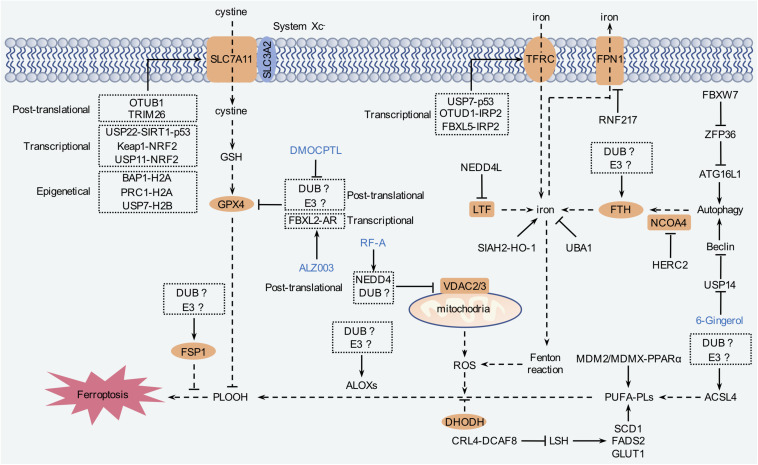
The regulation mechanism of ferroptosis by ubiquitination. Ferroptosis is tightly relevant to amino acid, iron, and lipid metabolism. Intracellular labile iron is capable of triggering lipid peroxidation to induce ferroptosis. System Xc^–^ transporter-mediated cystine uptake, which, in concert with GPX4, can reduce the cytotoxic lipid peroxides and inhibit ferroptosis. In addition, FSP1 and DHODH inhibit ferroptosis independent of GPX4. Modulation of these pathways by ubiquitination contributes to ferroptosis regulation.

Ubiquitination is a crucial step consisting of vast cellular processes, such as cell proliferation, differentiation, and death (Lu et al., 2021). Protein ubiquitination is mediated by a cascade of reactions carried out by E1 (ubiquitin-activating enzymes), E2 (ubiquitin-conjugating enzyme), and E3 (ubiquitin ligases) coordinately (Lu et al., 2021). Similarly to other post-translational modifications, ubiquitination is also reversible, termed as deubiquitination, which is conducted by DUBs (Harrigan et al., 2018; Sun et al., 2020). Dysregulated ubiquitination contributes to carcinogenesis as well as other diseases. Currently, accumulated evidence has emphasized that ubiquitination is pivotally involved in ferroptosis (Harrigan et al., 2018; Rape, 2018; Figure 5 and Table 1). At present, however, the role of ubiquitination still remains as a tip of the iceberg of ferroptosis.

**TABLE 1:** Ferroptosis regulation by E3s and deubiquitylating enzymes (DUBs).

Targets	Levels	E3s	DUBs	Biological functions	References
SLC7A11	Post-translation	TRIM26		Degrading SLC7A11; promoting ferroptosis	Zhu et al., 2021
			OTUB1	Stabilizing SLC7A11; suppressing ferroptosis	Liu T. et al., 2019; Chen S. et al., 2021
	Transcription		USP22	Increasing SLC7A11 expression; suppressing ferroptosis	Ma et al., 2020
	Epigenetic		BAP1	Suppressing SLC7A11 expression; promoting ferroptosis	Zhang et al., 2018
		PRC1		Suppressing SLC7A11 expression; promoting ferroptosis	Zhang et al., 2019a
			USP7	Suppressing SLC7A11 expression; promoting ferroptosis	Wang Y. et al., 2019
GPX4	Post-translation	Unknown		Degrading GPX4; promoting ferroptosis	Yang L. et al., 2020; Ding et al., 2021
	Transcription	FBXL2		Suppressing GPX4 expression; promoting ferroptosis	Chen et al., 2020
VDAC2/3	Post-translation	NEDD4		Degrading VDAC2/3; suppressing ferroptosis	Yang Y. et al., 2020
NRF2	Post-translation		USP11	Stabilizing NRF2; suppressing ferroptosis	Meng et al., 2021
		KEAP1		Degrading NRF2; promoting ferroptosis	Kansanen et al., 2013
NCOA4	Post-translation	HERC2		Degrading NCOA4; suppressing ferroptosis	Mancias et al., 2015
Beclin1	Post-translation		USP14	Deubiquitylating Beclin1; suppressing ferroptosis	Tsai et al., 2020
ATG16L1	Transcription	FBXW7		Promoting ATG16L1 expression; promoting ferroptosis	Zhang et al., 2020
TFRC	Transcription		USP7	Promoting TFRC expression; Promoting ferroptosis	Tang L.J. et al., 2021
FPN1	Post-translation	RNF217		Degrading FPN1;promoting ferroptosis	De Domenico et al., 2007; Jiang et al., 2021
IRP2	Post-translation	FBXL5		Degrading IRP2; suppressing ferroptosis	Dixon et al., 2012
			OTUD1	Stabilizing IRP2; promoting ferroptosis	Song et al., 2021b
LTF	Post-translation	NEDD4L		Degrading LTF; suppressing ferroptosis	Wang et al., 2020b
LSH	Post-translation	CRL4- DCAF8		Degrading LSH; promoting ferroptosis	Huang et al., 2020
HO-1	Post-translation	SIAH2		Degrading HO-1; suppressing ferroptosis	Chillappagari et al., 2020
	Transcription	SIAH2		Suppressing HO-1 expression; suppressing ferroptosis	Chillappagari et al., 2020

*E3 ubiquitin ligases and DUBs are pivotally involved in ferroptosis through targeting multiple substrates, including solute carrier family 7 member 11 (SLC7A11), voltage-dependent anion channels (VDAC2/3), nuclear factor, erythroid 2-like 2 (NRF2), and iron regulatory protein 2 (IRP2).*

### Ubiquitination of SLC7A11

System Xc^–^ is a disulfide-linked heterodimer composed of SLC7A11 and SLC3A2 subunits. SLC7A11 predominately confers ferroptosis-resistance and is highly expressed in many cancers (Koppula et al., 2020). SLC7A11 is indispensably involved in cystine-uptake, which is required for GSH formation (Koppula et al., 2020). Although many studies have focused on transcriptional regulation of SLC7A11, whether the post-translational modifications occur on SLC7A11 (especially ubiquitination) remains largely unknown. Liu et al. have revealed that OTU deubiquitinase ubiquitin aldehyde-binding 1 (OTUB1) physically binds SLC7A11, promoting its deubiquitination to stabilize SLC7A11 protein (Liu T. et al., 2019). Genomic depletion of *OTUB1* gene dramatically downregulates SLC7A11 expression and sensitizes cancer cells to ferroptosis. Indeed, OTUB1 is frequently overexpressed in multiple types of cancer, and OTUB1 deficiency abolishes xenograft growth in mice, which can be rescued by SLC7A11 overexpression. It has been reported that CD44, a cancer stem cell marker, positively regulates OTUB1-SLC7A11 pathway and promotes SLC7A11 protein stability for tumor growth (Liu T. et al., 2019). Moreover, endogenous hydrogen sulfides regulate SLC7A11 stability through persulfidation of OTUB-C91 in colon cancer cells (Chen S. et al., 2021). A recent study has displayed that tripartite motif-containing protein 26 (TRIM26) interacts with SLC7A11 and mediates its ubiquitination. In addition, TRIM26 overexpression promotes ferroptosis in hepatic stellate cells (HSCs) and suppresses CCl_4_-induced liver fibrosis (Zhu et al., 2021).

The tumor suppressor gene BRCA1-associated protein 1 (BAP1) encodes a nuclear DUB to reduce histone 2A ubiquitination (H2A-ub) on chromatin (Louie and Kurzrock, 2020). Zhang et al. (2018) have uncovered that BAP1 decreases H2A-ub occupancy on the SLC7A11 promoter to repress SLC7A11 expression and cystine uptake in a deubiquitinating-dependent manner, causing an elevation of lipid peroxidation and ferroptosis. Moreover, polycomb repressive complex 1 (PRC1) is a well-known E3 ubiquitin ligase of H2A-ub. A previous study has reported that PRC1 can enhance H2A-ub binding on SLC7A11 promoter, and PRC1 deficiency increases the protein level of SLC7A11, suggesting that the dynamic regulation of H2A ubiquitination importantly impacts on SLC7A11 expression (Zhang et al., 2019a).

A recent research has exhibited that p53 has a role in repressing SLC7A11 expression (Jiang et al., 2015). Notably, only p53 homozygous-deficient cells, but not the classical acetylation-defective mutant, show an increase in SLC7A11 expression. As a result of de-repression of SLC7A11, cystine uptake is dramatically increased accompanied by ferroptosis resistance. Meanwhile, SLC7A11 can be recognized by H2B-ub (mono-ubiquitination of histone H2B) via targeting lysine 120 (Wang Y. et al., 2019). The level of H2B-ub is decreased in cells ongoing ferroptosis. Loss of H2B-ub significantly enhances vulnerability of cells to ferroptosis. It should be noted that p53 has been shown to promote the translocation of USP7 inward in the nucleus, which has a role in de-ubiquitinating H2B. As a result of repressed SLC7A11 expression, the ferroptosis will be promoted (Wang Y. et al., 2019).

Ferroptosis is also involved in myocardial ischemia–reperfusion (MI/R) injury (Wu et al., 2021). Ma et al. (2020) have revealed an increase in p53 protein level, but downregulations of USP22, SLC7A11, and SIRT1 in response to MI/R injury. In this study, the authors have uncovered that deubiquitination and stabilization of SIRT1 by USP22 can repress transcriptional activity of p53, which leads to SLC7A11 upregulation and ferroptosis resistance. The expression of USP22 shows a protective effect against MI/R injury through SIRT1/p53/SLC7A11 axis *in vivo*. All these studies highlight the essential role of ubiquitination effect on the expression of SLC7A11 and ferroptosis inhibition.

### Ubiquitination of GPX4

GPX4 is an essential selenoprotein reducing phospholipid hydroperoxide and plays a key role in defending cells against lipid peroxidation (Friedmann Angeli and Conrad, 2018; Forcina and Dixon, 2019) and ferroptosis (Hassannia et al., 2019). Inhibition of GPX4 by a synthesized small molecule induces cell lethality and lipid peroxidation (Yang et al., 2014; Gaschler et al., 2018). However, whether a modification of GPX4 at the post-translational level exists is still largely unknown. Androgen receptor (AR) is a steroid hormone receptor overexpressed in several types of cancer (Narayanan, 2020) and is inversely correlated with survival rate (Chen et al., 2020). Chen et al. (2020) has revealed that ALZ003, an FDA-approved curcumin analog drug, induces AR degradation via FBXL2-mediated ubiquitination. Notably, AR expression is also important for redox homeostasis. Moreover, either AR knockdown or ALZ003 treatment dramatically increases the level of lipid ROS followed by a decrease in the protein level of GPX4 in glioblastoma cells.

The natural product parthenolide (PTL) has attracted much attention due to its anticancer effect (Sztiller-Sikorska and Czyz, 2020). However, the clinical application of PTL remains to be investigated because of low oral bioavailability and poor solubility (Araújo et al., 2020). A derivative of PTL, DMOCPTL, has been designed with an improvement of solubility. DMOCPTL is capable of repressing the growth of triple negative breast cancer (TNBC) cells. Lipid ROS and iron level are significantly increased upon DMOCPTL treatment in TNBC, while the GPX4 protein level is reduced. Mechanistically, DMOCPTL can bind to GPX4 and promote its ubiquitination in TNBC cells. DMOCPTL effectively inhibits breast tumor growth and prolongs survival rate in mice (Ding et al., 2021). Palladium pyrithione complex (PdPT), a broad-spectrum DUB (including USP7, USP10, USP14, USP15, USP25, and UCHL5) inhibitor, can also cause GPX4 protein degradation in non-small cell lung cancer cells (Yang L. et al., 2020). As mentioned above, we propose that ubiquitination modification of GPX4 drives the vulnerability to ferroptosis. However, the specific E3 ubiquitin ligases and DUBs for GPX4 remain to be identified.

### Ubiquitination of Voltage-Dependent Anion Channel2/3

Voltage-dependent anion channels (VDACs) are located at the outer membrane of the mitochondrion allowing shuttling of metabolites and ions between the mitochondrion and cytosol (Fang and Maldonado, 2018). Erastin can target VDAC2/3 in addition to SLC7A11 and causes VDAC2/3 degradation. Yang Y. et al. (2020) have reported that treatment of erastin is able to elevate the expression of neural precursor cell-expressed developmentally downregulated protein 4 (NEDD4), which is an E3 ubiquitin ligase. Genomic deletion of *NEDD4* increases expression level of VDAC2/3 and enhances ferroptosis susceptibility. Additionally, natural metabolite biflavonoids extracted from plants are regarded as promising anticancer drugs in breast cancer treatment. The C-3′-C-6″ type of biflavonoids robustaflavone A (RF-A) extracted from *Selaginella trichoclada* has been shown to decrease cell viability of breast cancer and diminish the NEDD4 expression. Eventually, VDAC2 is stabilized accompanied by lipid peroxidation and ferroptosis (Xie et al., 2021).

### Ubiquitination of Nuclear Factor, Erythroid 2-Like 2

The transcription factor NRF2 plays a vital role in ferroptosis and cancer progression (Dodson et al., 2019). It has been widely reported that E3 ligase KEAP1 promotes NRF2 ubiquitination (Kansanen et al., 2013), whereas the deubiquitination mechanism of NRF2 remains largely elusive. Meng et al. (2021) have identified USP11, which can work as a DUB of NRF2. A stabilized NRF2 protein has been revealed to be attributed to USP11-modified deubiquitination. Functionally, USP11 deficiency contributes to the induction of ferroptosis, which can be rescued by NRF2 expression. USP11 is highly expressed in lung cancer patients and correlates to poorer prognosis. These studies demonstrate that DUBs play a pivotal role in the modulation of ferroptosis by regulating ubiquitination of ferroptosis-related proteins.

### Ubiquitination in Autophagy

Autophagy-mediated ferritin degradation (ferritinophagy) is an essential step involved in ferroptosis (Gao et al., 2016). NCOA4 is a selective cargo receptor for the autophagic turnover of ferritin, a process critical for regulation of intracellular iron bioavailability. The arginine residues in FTH1 and a C-terminal element in NCOA4 are essential for ferritin degradation in autophagosomes. Moreover, NCOA4 stability is under the control of the ubiquitin proteasome system in addition to autophagy. Ubiquitin-dependent NCOA4 turnover is accelerated by excessive iron, which is associated with HECT domain and RCC1-like domain 2 (HERC2) ubiquitin ligase (Mancias et al., 2015). HERC2 only binds to NCOA4 when the iron level is increased, which leads to NCOA4 degradation by the proteasome. When the concentration of iron becomes lower, the interaction between HERC2 and NCOA4 does not take place. Therefore, an increase in the protein level of NCOA4 will promote ferritinophagy, which subsequently enlarges labile iron poor and induces ferroptosis in the cells. Furthermore, autophagy-related proteins are not only essential for autophagy induction but also are involved in erastin-induced ferroptosis (Zhou et al., 2020). ATG7 plays a central role in both autophagy-specific UBL systems (Hong et al., 2011) and ferroptosis (Hou et al., 2016). ATG7 can work as an E1 enzyme for ubiquitin-like proteins (UBL), such as ATG8 and ATG12. ATG7 enables ATG12 and ATG8 targeting their molecules by binding to them and motivating their transfer to an E2 enzyme (Kaiser et al., 2013).

In addition, Beclin1 and 6-Gingerol have been shown to regulate autophagy and ferroptosis mediated by USP14, which can be suppressed by 6-Gingerol. Mechanistically, USP14 affects autophagy through deubiquitination of Beclin1. Moreover, administration of 6-Gingerol represses tumor growth followed by increased intracellular iron level and ferroptosis. Thus, 6-Gingerol may be utilized as a therapeutic agent to promote ferroptosis in lung cancer treatment (Tsai et al., 2020).

Hepatic stellate cell (HSC) plays an important role in liver fibrosis. Targeting HSCs is considered a potent approach for liver fibrosis alleviation (Tsuchida and Friedman, 2017). However, overexpression of RNA-binding protein ZFP36 (also known as TTP) shows resistance to ferroptosis in HSCs (Zhang et al., 2020). Zhang et al. (2020) have showed that ZFP36 inhibits autophagy by destabilizing autophagy-related 16-like 1 (ATG16L1) mRNA, which is required for autophagy induction. A decrease in stability of ATG16L1 mRNA abolishes ferritinophagy–associated ferroptosis. In addition, other studies also reveal that erastin can promote ZFP36 degradation via the E3 ubiquitin ligase FBXW7. FBXW7 is able to identify ZFP36 through the consensus degron (SFSGLPS). Due to the fact that effective therapy for liver fibrosis has not been approved yet, thus far, ferroptosis may be utilized as a novel approach to overcome liver fibrosis.

### Ubiquitination in Iron Metabolism

TFRC is a major iron importer and takes the responsibility of ferric iron uptake (Yan et al., 2020). Moreover, increased intracellular iron has been regarded as a surrogated marker of ferroptosis (Yu et al., 2019). Tang L.J. et al. (2021) have found evidence of ubiquitination effect compromised in TFRC expression, which is enhanced by USP7-mediated p53 stabilization. The small molecular inhibitor targeting USP7 decreases ferroptosis. Therefore, the USP7/53/TFRC axis appears to be a potential target for myocardial I/R injury therapy. FPN1 is the sole iron exporter existing on the plasma membrane. It has been displayed that FPN1 is able to be phosphorylated and ubiquitinated (De Domenico et al., 2007); however, the corresponding E3 ubiquitin ligases and DUBs remain largely unknown. A recent study has shown that RNF217 mediates the ubiquitination and subsequent degradation of FPN1 (Jiang et al., 2021).

Iron regulatory protein 2 (IRP2) plays a central role in iron metabolism. FBXL5 has been identified as the E3 ubiquitin ligase targeting on IRP2 for its degradation (Vashisht et al., 2009). Importantly, FBXL5 silencing stimulates an increase in hepatocellular iron level and embryonic lethality (Moroishi et al., 2011) as well as ferroptosis (Dixon et al., 2012). It has been also reported that OTUD1 promotes TFRC-mediated iron transport through deubiquitinating of IRP2 irrespective of iron concentration, eventually leading to ferroptosis (Song et al., 2021b).

Wang et al. (2020b) have screened 571 ubiquitin-related genes, which are potentially related to ferroptosis regulation. The E3 ligase NEDD4 like E3 ubiquitin protein ligase (NEDD4L), a novel ferroptosis suppressor in human pancreatic cancer cells, has been found with a role in regulating iron metabolism by targeting iron-binding transport protein lactotransferrin (LTF).

Ubiquitin-like modifier-activating enzyme 1 (UBA1) is a potential marker for hepatocellular carcinoma (HCC) prognosis since its expression positively correlates with survival rate. Inhibition of UBA1 reduces proliferation, migration, and invasion in HCC cells. Moreover, both iron concentration and malondialdehyde (MDA) levels are elevated in response to UBA1 suppression. The expression of UBA1 has been found related to NRF2 as well as the downstream targets like heme oxygenase-1 (HO-1), NAD(P)H quinone dehydrogenase 1 (NQO1), and FTH1, which are associated with iron metabolism regulation. These findings suggest that UBA1 may play a vital role in ferroptosis (Shan et al., 2020).

Epigenetic factor lymphoid-specific helicase (LSH) is a member of SNF2 family chromatin remodeling ATPases (Baumann et al., 2020; Ni et al., 2020). The expression of LSH is essential for an adequate maintenance of genomic stability (Ni et al., 2020). Dysregulation of LSH has been exhibited in various malignancies (Liu and Tao, 2016; Yang et al.,2019a,b; He et al., 2020) as well as chronological aging (Zhou et al., 2009). Intriguingly, LSH also inhibits ferroptosis by sequestrating labile iron and limits the generation of lipid ROS (Jiang et al., 2017). Moreover, LSH has been uncovered to be associated with WD40-repeat protein 76 (WDR76) to inhibit ferroptosis by activating lipid metabolism-associated genes, including glucose transporter 1 (GLUT1), ferroptosis-related gene stearoyl-CoA desaturase 1 (SCD1), and fatty acid desaturase 2 (FADS2) (Jiang et al., 2017). It has been reported that erastin induces LSH destabilization, and CRL4–DCAF8 synergizes with WDR76 to control the protein levels of LSH (Huang et al., 2020). E3 ligase CRL4–DCAF8 mediates polyubiquitination and proteasomal degradation of LSH, and WDR76 antagonizes DCAF8-targeted LSH proteolysis through competitive inhibition of the holo–CRL4–DCAF8–LSH complex assembly (Huang et al., 2020).

Heme oxygenase-1 (HO-1) catalyzes the oxidative cleavage of heme to biliverdin, iron, and carbon monoxide. The role of HO-1 in erastin-induced ferroptosis has been investigated (Chillappagari et al., 2020). A recent study has identified seven *in absentia* homologs (SIAH2) as crucial E3 ubiquitin ligases to control HO-1 protein stability. Controversially, SIAH2 can also downregulate transcriptional expression of HO-1, which depends on the transcription factor NRF2 (Chillappagari et al., 2020). Thus, SIAH2 can govern the expression level of HO-1 by a dual mechanism.

### Ubiquitination in Lipid Metabolism

Generation of lipid peroxides determines the sensitivity of ferroptosis. Thus, lipogenesis has been considered as a potent strategy to defend against ferroptosis in cancer cells. E3 ubiquitin ligases MDM2 and MDMX are well-known negative regulators of p53. It has been reported that MDM2 and MDMX can promote ferroptosis via PPARα-mediated lipid remodeling irrespective of p53 activity. Additionally, MDM2 or MDMX depletion can also lead to increased levels of FSP1 and CoQ10, which are potent suppressors of ferroptosis (Venkatesh et al., 2020).

### Ubiquitination in Hippo Pathway

YAP and TAZ are the main downstream factors regulated by NF2. Activation of YAP enhances expression of iron transporter, TFRC (Wu et al., 2019), followed by increased level of intracellular iron and ferroptosis induction in mesothelioma cells. YAP also promotes ferroptosis via different targets such as ACSL4. Interestingly, TAZ, but not YAP, appears to specifically sensitize renal cell carcinoma cell lines to ferroptosis via regulation of epithelial membrane protein 1 (EMP1), suggesting a context-dependent role of YAP/TAZ in ferroptosis (Yang et al., 2019c). The E3 ubiquitin ligase FBXW7 has been regarded as a tumor suppressor. A recent study has shown that FBXW7 targets YAP for degradation (Tu et al., 2014). However, FBXW7 can induce ferroptosis by targeting RNA-binding protein ZFP36/TTP (Zhang et al., 2020). These studies indicate that FBXW7 plays a crucial role in ferroptosis regulation. However, further studies are necessary to address more details under different contexts. β-transducin repeat-containing E3 ubiquitin protein ligase (βTrCP) targets YAP and TAZ for degradation by the SCF ubiquitin ligase complex. Recently, deletion of βTrCP has been shown to inhibit erastin-induced ferroptosis in lung cancer cells (Zhang X. et al., 2021). However, the mechanisms remain to be further elucidated yet.

## Targeting Ubiquitination for Ferroptosis in Cancer Therapy

Multiple studies have revealed that induction of ferroptosis is a promising approach in cancer therapy (Cramer et al., 2017; Zhang et al., 2019b; Badgley et al., 2020; Chen et al., 2021a; Koren and Fuchs, 2021). However, most synthetic ferroptosis inducers appear to be unsuitable for clinical application, thus far, due to the poor solubility and *Ki* value *in vivo* (Shen et al., 2018; Gautheron et al., 2020). Besides, an adequate druggable candidate involved in ferroptosis has not been uncovered yet (Dang et al., 2017). As accumulated evidence has shown that ubiquitination plays a vital role in ferroptosis, targeting the ubiquitin system will be an alternative strategy to further realize the role of ferroptosis in cancer and other diseases. Notably, the FDA-approved 20S proteasome inhibitors bortezomib and carfilzomib have been used for the treatment of hematological malignancies (Farshi et al., 2015; Nunes and Annunziata, 2017; Chari et al., 2019). Inhibition of the proteasome by bortezomib has been validated for targeting the UPS in cancer therapy (Crawford and Irvine, 2013; Ettari et al., 2018); however, the acquired resistance to bortezomib always occurred in clinical settings. Palladium pyrithione complex (PdPT) targeting upstream components of the proteasome has been investigated to enhance the anticancer effect of bortezomib by targeting GPX4 degradation and induce both ferroptosis and apoptosis (Yang L. et al., 2020). Carfilzomib is a second-generation proteasome inhibitor approved by the FDA. Interestingly, iron has been shown to improve carfilzomib efficacy in MM cells suggesting that a combination of iron supplementation and ferroptosis induction may represent a novel strategy to overcome resistance to carfilzomib (Bordini et al., 2020). The selenoprotein thioredoxin reductase 1 (TXNRD1) plays a vital role in protecting tumor cells against oxidative stress. Modulation of ferroptosis sensitivity by TXNRD1 has been addressed in pancreatic cancer cells (Cai et al., 2020). It should be noted that lenalidomide, which has been shown to interact with the ubiquitin E3 ligase cereblon, has been approved for medical use since 2005. Lenalidomide is capable of inhibiting TXNRD1 that leads to an accumulation of cytotoxic H_2_O_2_ levels, suggesting that lenalidomide may have roles in ferroptosis regulation (Sebastian et al., 2017).

Targeted protein ubiquitination and subsequent degradation using the Proteolysis Targeting Chimeras (PROTACs) have emerged as a novel therapeutic technology in drug discovery (Paiva and Crews, 2019). In 2019, PROTAC ARV-110, which targets the androgen receptor (AR) for degradation in prostate cancer, has been approved by the FDA for phase I clinical trials (Neklesa et al., 2017; Pettersson and Crews, 2019; Schapira et al., 2019; Ding et al., 2020; Poh, 2020; Wang et al., 2020a). Development of novel therapies by targeting the ubiquitin system (DUBs/E3s inhibitors or PROTACs) on ferroptosis-related proteins may facilitate the clinical application of ferroptosis in the future (Popovic et al., 2014; Liu J. et al., 2021; Zhou and Sun, 2021).

## Conclusion and Perspective—Next Decades

Although emerging evidence has pointed out the potential role of ubiquitination in ferroptosis, the details of the mechanism remain to be elucidated. Identification of specific enzymes involved in ubiquitination of ferroptosis will solidify the understanding of the role of ferroptosis in cancers as well as in other disorders. Still, the specific E3 ubiquitin ligases for targeting GPX4, FSP1, and other essential proteins in ferroptosis are still unknown. Whole genome-wide or sub-pool of E3/DUBs library CRISPR-cas9 screening approach will be of great help to identify key ubiquitination regulators in ferroptosis. Thus far, most ferroptosis-related research predominantly focuses on cultured cells and xenograft models in nude mice; however, the precise regulations of ferroptosis in physiological and pathological conditions are unclear. Elucidation of the relationship between ubiquitination and ferroptosis will provide novel insights into cancer therapy.

SLC7A11 overexpresses in most types of cancer and is regulated by multiple transcriptional factors (Ye et al., 2014; Jiang et al., 2015; Zhang et al., 2018; Ogiwara et al., 2019). However, whether post-translational modification, especially ubiquitination, contributing to this overexpression (in addition to OTUB1 and TRIM26) is unclear. Mass spectrometry (MS) data shows that SLC7A11 can be highly ubiquitinated at multiple sites (PhosphoSitePlus) (Hornbeck et al., 2015), emphasizing the importance of E3 ubiquitin ligase in SLC7A11-mediated ferroptosis resistance. GPX4 holds the core fortress in ferroptosis. GPX4 is a selenoprotein, which contains selenocysteine, a non-canonical amino acid coded by the termination codon (UGA). A selenocysteine insertion sequence (SECIS) of the selenoprotein mRNA is necessary for the translation of UGA to Sec, via a series of precise protein collaborations (Driscoll and Copeland, 2003). CRL2 ubiquitin ligase, a member of the cullin-RING ligase (CRL) superfamily, specifically eliminates truncated proteins produced by failed UGA/Sec decoding, controlling selenoprotein quality (Lin et al., 2015). A recent study has revealed that peroxisome proliferator-activated receptor γ (PPARγ) can act as an E3 ubiquitin ligase, mediating ubiquitination and degradation of selenoproteins (SelS and SelK) (Lee et al., 2019). Notably, rapamycin, an mTOR inhibitor, induces GPX4 protein degradation at high doses in human pancreatic cancer cell lines (Liu Y. et al., 2021). However, the mechanism of GPX4 degradation is still unclear, especially that the specific E3 ligase and DUB remain to be identified. This may be achieved by identifying E3/DUB interaction partners of these substrates via MS strategy or Bioplex database^1^ in addition to E3/DUB prediction tools such as UbiBrowser.^2^

## Author Contributions

XW, YW, and ZL wrote the manuscript. JQ and PW edited the manuscript. All authors contributed to the article and approved the submitted version.

## Conflict of Interest

The authors declare that the research was conducted in the absence of any commercial or financial relationships that could be construed as a potential conflict of interest.

## Publisher’s Note

All claims expressed in this article are solely those of the authors and do not necessarily represent those of their affiliated organizations, or those of the publisher, the editors and the reviewers. Any product that may be evaluated in this article, or claim that may be made by its manufacturer, is not guaranteed or endorsed by the publisher.

## Footnotes

1
https://bioplex.hms.harvard.edu

2
http://ubibrowser.ncpsb.org.cn/ubibrowser/
